# Dopamine D1-signaling modulates maintenance of functional network segregation in aging

**DOI:** 10.1016/j.nbas.2023.100079

**Published:** 2023-06-15

**Authors:** Robin Pedersen, Jarkko Johansson, Alireza Salami

**Affiliations:** a Department of Integrative Medical Biology, Umeå University, Umeå, Sweden; b Umeå Center for Functional Brain Imaging (UFBI), Umeå University, Umeå, Sweden; c Wallenberg Center for Molecular Medicine (WCMM), Umeå University, Umeå, Sweden; d Aging Research Center, Karolinska Institutet & Stockholm University, Stockholm, Sweden

**Keywords:** Dopamine, D1DR, Functional connectivity, Dedifferentiation, Aging, Working memory

## Abstract

Past research has shown that as individuals age, there are decreases in within-network connectivity and increases in between-network connectivity, a pattern known as functional dedifferentiation. While the mechanisms behind reduced network segregation are not fully understood, evidence suggests that age-related differences in the dopamine (DA) system may play a key role. The DA D1-receptor (D1DR) is the most abundant and age-sensitive receptor subtype in the dopaminergic system, known to modulate synaptic activity and enhance the specificity of the neuronal signals. In this study from the DyNAMiC project (N = 180, 20-79y), we set out to investigate the interplay among age, functional connectivity, and dopamine D1DR availability. Using a novel application of multivariate Partial Least squares (PLS), we found that older age, and lower D1DR availability, were simultaneously associated with a pattern of decreased within-network and increased between-network connectivity. Individuals who expressed greater distinctiveness of large-scale networks exhibited more efficient working memory. In line with the maintenance hypotheses, we found that older individuals with greater D1DR in caudate exhibited less dedifferentiation of the connectome, and greater working memory, compared to their age-matched counterparts with less D1DR. These findings suggest that dopaminergic neurotransmission plays an important role in functional dedifferentiation in aging with consequences for working memory function at older age.

## Introduction

As individuals grow older, various structural and functional changes occur in the brain, with implications for several cognitive and behavioral abilities[25], [35], [75], [89]. A prominent aspect of brain aging are alterations in the degree of synchronous activity between brain regions, referred to as functional connectivity (FC). Several studies have reported age-related decline in FC within highly synchronous networks of brain regions, such as the default mode network (DMN)[2], [26], [78], [88], [90]and the fronto-parietal network[4], [12], [13], [26]. There is also evidence for age-related increases in FC between networks[14], [16], [29], [87]. The observation of weaker within- and greater between-network connectivity in aging has led to the notion of neural dedifferentiation (for review, see[44]), originally referring to reduced specificity in task-related brain activation at older age[19], [66], [92]. While there is ample evidence of age-related dedifferentiation of the brain’s functional network structure[15], [27], [56], [67], the underlying mechanisms of these changes are poorly understood. A likely contributing factor is the degree of white-matter fiber integrity[2], [5], [67], although the relationship between brain structure and function is typically modest[24], [54]and varies across different brain regions[11], [91], [95]. In fact, recent evidence suggests that associative cortical regions, which is the most age-sensitive to functional alterations, show the least structural–functional correspondence[91], with relatively stable correspondence across the life span[96]. Alternatively, it has been proposed that neural dedifferentiation may be a consequence of reduced neural efficiency related to neurochemical shifts in ascending neuromodulatory systems[49], [50], [51], [57].

Catecholamines, such as dopamine (DA), have been found to affect the signal-to-noise ratio of neural transmission[22], [82], [84]. Insufficient modulation may therefore lead to greater variability of neural signaling across the central nervous system[31], [49], [50]. DA is particularly interesting in this regard, since it has been linked to multiple physiological functions including motor control, reward mechanisms, reinforcement learning, and higher-order cognitive functions[1], [6], [73], [76], [81]. Importantly, measures of DA D1-receptor (D1DR) and D2-receptor (D2DR) availability decline markedly in aging[36], [39], [41], [72], [83]and serve as strong mediators of age-related cognitive decline [6], [7], [9]. In line with the notion of brain maintenance[64], [99], preserved integrity of the dopaminergic system has been linked to better cognitive performance in older age[71], [98]. Moreover, DA receptor-expression shares organizational principles with brain function [34], [65], [94], suggesting that the distinctiveness of functional brain systems may be modulated by the integrity of the dopaminergic system[70], [71], [74]. However, no study to date have tested whether DA receptor availability mediate age-related dedifferentiation in functional architecture.

In this study from the DyNAMiC project (N = 180, 20–79 years,[62], we use functional magnetic resonance imaging (fMRI) and Positron Emission Tomography (PET) with [^11^C] SCH23390 radioligand to investigate the relationship between age, D1DR availability, and FC measured during resting state. The DA D1-receptor was selected since it is among the most age-sensitive dopaminergic marker[36], [41]. Specifically, we focus on D1DR availability in caudate due to its extensive dopaminergic innervations and connectivity to both task-positive (e.g., frontoparietal) and task-negative (DMN) networks[70], [101]. Importantly, caudate D1DR have been shown to be the most age-sensitive DA-rich region[37], with well-established link to measures of functional connectivity and cognition[6], [18], [20], [43], [48], [63], [70].

We used multivariate Partial Least Squares (PLS)[58], [59] analysis to examine the effects of age and caudate D1DR for FC across 264 brain regions. If the effects of age and caudate D1DR on functional architecture are intercoupled, one would expect a single latent variable (LV), indicating that older age, and less D1DR, is related to decreased within- and increased between-network connectivity. Alternatively, D1DR may be linked to specific functional differences, unrelated to the pattern of age-related dedifferentiation in connectivity. Additional analysis investigate whether individual differences in age and D1DR-sensitive FC architecture have implications for cognition, particularly working memory function (c.f.[8], [10], [71], [74]). In line with the maintenance hypothesis (e.g.,[64]), we expected older individuals with greater D1DR availability to exhibit more youth-like functional architecutre and working memory performance compared to their age-matched counterparts.

## Methods

### Participants

The DyNAMiC study participants (*N* = 180) were recruited by random selection from the population registry of Umeå, Sweden, stratified by six age cohorts between 20 and 80 years (*n* = 30 per decade; 50% females). Participants were screened for a set of exclusion criteria, including contraindications to magnetic imaging, medical conditions or treatment potentially affecting brain function and cognition, neurological disorders, brain pathology, and cognitive impairment. All participants completed a full set of functional and structural MRI scans, and one hundred seventy seven participants completed [^11^C]SCH23390 PET scans. Exclusions were due to the following: one showed indications of subcutaneous injection, two were excluded due to technical problems, and one participant declined to undergo PET. Three participants were excluded due to unreliable D1DR estimates in caudate. The final sample for the current study included 173 participants (82 females) aged 20 – 78 years (mean = 50, SD = 17.37).

### PET imaging

The radiotracer [11C]SCH23390 was produced at the radiochemistry laboratory of Norrlands Universitestsjukhus, Umeå University, using previously described procedures[62]. Prior to the injection, a 5-minute low-dose helical CT scan was obtained for PET-attenuation correction. Continuous PET measurements were collected in list-mode format for 60 min, beginning at the time of injection. The list-mode data were offline re-binned to create a sequence of time-framed data with increasing frame lengths (6 × 10; 6 × 20; 6 × 40; 9 × 60; 22 × 120 s). Time-framed corrected PET images were reconstructed using the VUE Point HD-SharpIR algorithm. Pre-processing included frame-to-frame head motion correction and registration to T1-weighted MRIs using Statistical Parametric Mapping software (SPM12, Wellcome Institute, London, UK). Partial-volume-effect correction was performed using a symmetric geometric transfer matrix method implemented in FreeSurfer with an estimated point-spread function of 2.5 mm full-width-at-half-maximum (FWHM). Estimation of target binding potential (BP) to non-displaceable (BPND) binding was computed with the cerebellum as reference region[30]. Mean bilateral caudate BPND estimates were finally computed using a simplified reference tissue model (SRTM)[47], delineated by Freesurfer segmentations.

### MRI imaging

High-resolution anatomical T1-weighted images were acquired by a 3D fast spoiled gradient-echo sequence with the following parameters: 176 sagittal slices, thickness = 1 mm, repetition time (TR) = 8.2 ms, echo-time (TE) = 3.2 ms, flip angle = 12°, and field of view (FOV) = 250 × 250 mm. Anatomical T1-weighted images were used to parcellate subcortical structures using Freesurfer 6.0 (https://surfer.nmr.mgh.harvard.edu) [23], with manual corrections performed if necessary. Whole-brain functional images were acquired during rest. Subjects were instructed to keep their eyes open and let their minds wander while remaining as still as possible. Functional images were sampled using a T2*-weighted single-shot echo-planar imaging (EPI) sequence, with a total of 350 volumes collected over 12 min. The functional sequence was sampled with 37 transaxial slices; slice thickness = 3.4 mm, 0.5 mm spacing; TR = 2000 ms, TE = 30 ms, flip angle = 80°, and FOV = 250 × 250 mm.

The resting-state fMRI data were preprocessed using Statistical Parametric Mapping (SPM12: Wellcome Trust Centre for Neuroimaging, https://www.fil.ion.ucl.ac.uk/spm/) implemented in an in-house software. The functional images were first corrected for slice-timing differences and in-scanner motion, followed by registration to anatomical T1-images. Distortion correction was performed using subject-specific field maps that were co-registered to the anatomical T1-images. All volumes were temporally demeaned, and linear and quadratic effects were removed. A 36-parameter nuisance regression model was applied[17], including mean cerebrospinal, white-matter, and whole-brain signal in addition to six motion parameters, including the variables squares, derivatives, and squared derivatives. The nuisance model also included a set of spike regressors, defined as binary vectors of motion-contaminated volumes exceeding a volume-to-volume root-mean-squared (RMS) displacement of 0.25 mm[80]. A temporal band-pass filter (0.008 – 0.09 Hz) and nuisance regression was applied simultaneously as to not re-introduce nuisance signals[33]. Finally, the functional images were normalized to a group-representative template using Diffeomorphic Anatomical Registration using Exponentiated Lie algebra (DARTEL)[3]followed by affine transformation to MNI space (ICBM152NLin2009). Four individuals were excluded from the intermediate template-generation step due to non-pathological anatomical irregularities but included for all other analyses. To mitigate aliasing effects during normalization, the images were smoothed using a 6-mm FWHM Gaussian kernel[3].

### Graph construction

Mean resting-state timeseries were sampled from 264 cortical and subcortical brain regions (5-mm radius spheres) based on a commonly used functional connectivity parcellation[68]. Each brain region was labeled according to 14 resting-state networks defined by a consensus partition (see [68] for details about network assignments). To minimize sampling from non-gray matter voxels, each parcel was eroded by a liberal gray matter mask (voxels < 0.1% threshold were eroded). The sampled timeseries were subsequently correlated using Pearson's correlations followed by Fisher's r-to-z transformation to create a 264 × 264 adjacency matrix for each participant and coefficient along the main diagonals were set to zero.

### Working memory

Working memory was evaluated using three tasks: letter updating, number updating, and spatial updating. Full task descriptions have been reported elsewhere[62]. In brief, the letter updating task involved showing participants a sequence of letters and having them remember the last three. The task consisted of four practice trials and 16 test trials with either 7, 9, 11, or 13 letter sequences with a maximum number of correct answers of 48. The number updating task had a 3-back design, with three boxes displayed on the screen. Participants had to determine if the current number in a specific box matched the one shown three numbers before. There were two practice trials and four test trials with 30 numbers each, with a maximum score of 108. In the spatial updating task, three grids were displayed on the screen with a blue circular object placed at a random location in each. Participants mentally moved the objects according to arrows and then placed them in the correct square. This process was repeated, resulting in the objects moving two steps from their original location. Performance was calculated based on the sum of correct location indications across trials with a maximum score of 30. Summary scores from each task were standardized (mean = 50, SD = 10), and a composite score of working memory performance was computed as participants’ mean score across the three tasks.

### Statistical analyses

All statistical analyses were performed using MATLAB v.9.10.0 (R2021a). The code used for performing the analyses reported in the current study is publicly available at the following GitHub repository: https://github.com/robinpedersen/D1DR_FC_PLS.

### Partial least squares

Behavioral Partial Least Squares (bPLS)[59]is an analytical approach typically used to analyze associations between behavioral characteristics and functional brain activation[45]. Here, we use this analytical framework to examine the relationship between FC in relation to caudate D1DR availability and age. Therefore, the so-called *behavioral variables*, i.e., D1DR availability and age, will henceforth be referred to as *variables of interest*.

**Fig. 1:**
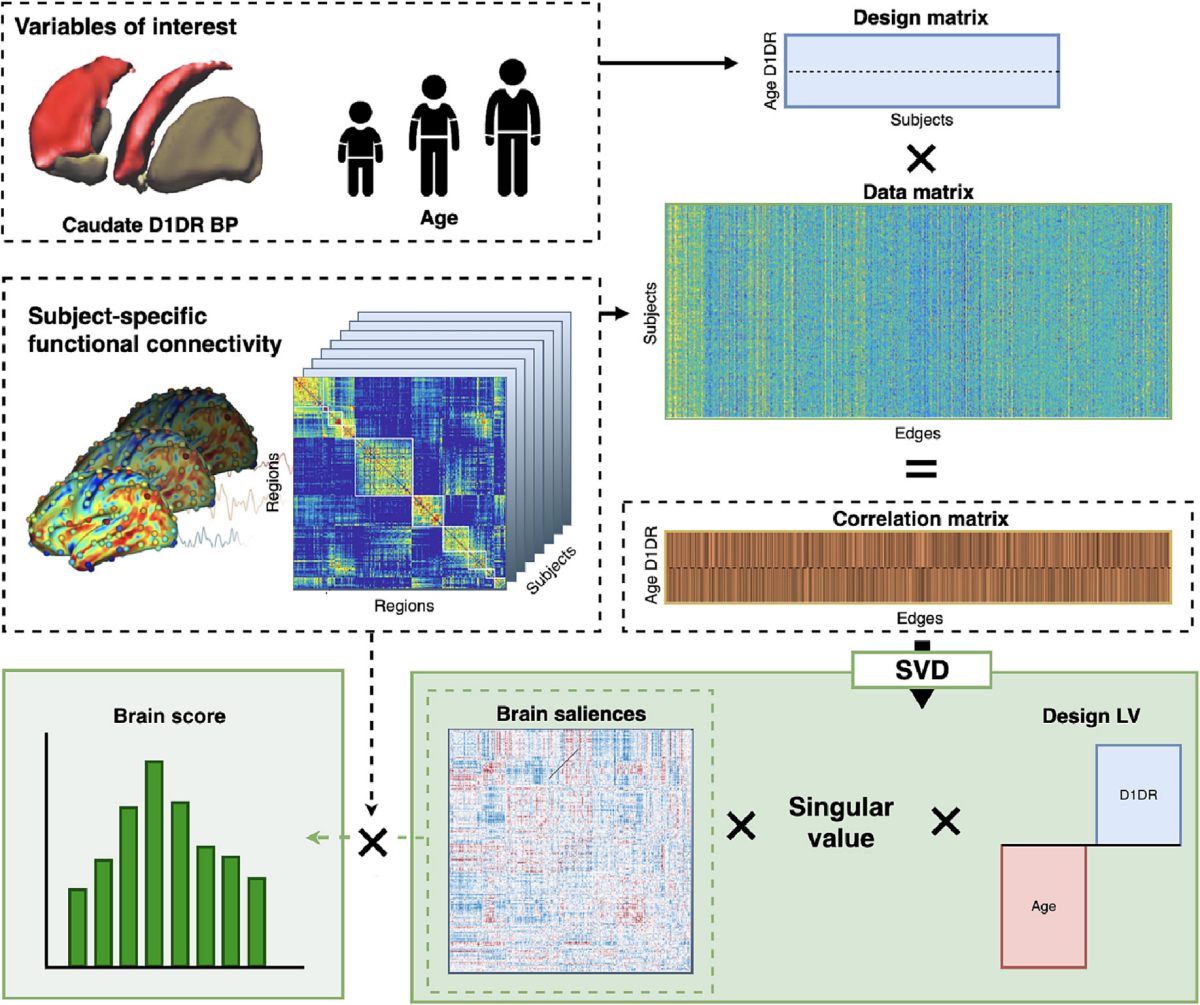
Overview of PLS analysis. The variables of interest (caudate D1DR availability and subjects’ age) are correlated with a data matrix of subjects’ FC edges. The resulting correlations are decomposed using SVD, yielding a left-singular vector of brain saliences, reshaped to the size of the original connectivity matrix. Significance of the LV is permutation tested on the singular value. The right-singular vector reflects a design LV, reflecting association strength between the variables of interests in relation to FC. Brain scores were computed as the dot product between brain saliences and subject-specific FC matrices.

The analytical approach of bPLS (Fig. 1) starts out by stacking the variables of interest in a design matrix, rows corresponding to subjects and columns to each variable of interest. Similarly, a data matrix is created with the same number of rows as the design matrix, with columns containing edge weights from subjects’ connectivity matrices. The two matrices are cross-correlated, yielding a variable-by-edges correlation matrix. The correlation matrix is subsequently decomposed by singular value decomposition (SVD). The resulting left singular vector is referred to as *brain saliences*, representing how well each edge relates to each LV across the sample. The right singular vector is referred to as a *design LV*, representing how well the variables of interest relate to the overall pattern of the LV. To quantify how well each subject’s connectome is represented by the pattern of brain saliences, subject-specific *brain scores* were computed as the dot product between brain saliences and subjects’ connectivity matrices. Brain scores therefore represent how well each subject’s connectivity profile is characterized by the spatial pattern of brain saliences.

Significance of associations between the brain saliences and the variables of interest were assessed by 1000 permutation tests on the singular value. Robustness of brain saliences was evaluated by bootstrapping, resampling the data 1000 times with replacement. The robustness of each edge’s brain salience was subsequently defined by *bootstrap ratios* (BSR), obtained by dividing brain saliences by their bootstrapped standard error, such that greater absolute BSR indicate greater robustness[79]. The distribution of BSRs is approximately equivalent to a z-distribution[21]. Significance was therefore determined by absolute BSRs>2.8 (*p* ≈ 0.005).

The same model was fitted to an alternative set of functional data to evaluate potential effects related to global signal regression (GSR) during data preprocessing. The pipeline without GSR yielded qualitatively similar results, with no statistical difference between permutated singular values (overlapping 95% CIs).

### System segregation

To evaluate whether individual differences in FC is differentially associated with age and D1DR availability within and between functional networks, we compared subject-specific brain scores with a commonly used metric of system segregation[16], [67]. The segregation metric, reflecting the ratio of connectivity within networks in relation to the connectivity between networks, was computed by subtracting mean within-network connectivity from between mean within-network connectivity, divided mean within-network connectivity for each subject.

## Network level connectivity associations

To quantify the network-level contributions to the connectivity pattern identified by the PLS analysis, we used a permutation method described in previous work[38], [60]. In brief, we first stratified the BSR matrix by positive and negative PLS weights. The two matrices were binarized, setting positive and negative weights to 1 and −1, respectively. Non-significant edges (|BSR| < 2.8) were set to zero. The network-level effects were quantified by averaging all weights for each network respectively, yielding a 14 × 14 network matrix[68]. Next, network-average weights were permutation tested by repeating the procedure on the full thresholded BSR matrix, re-ordering the network labels but preserving the number of nodes originally assigned to each network. New network means were computed 1000 times for the reordered node-network labels to build a null-distribution of network averages for the positive and negative weight-matrices separately. Significance of network-level PLS effects determined by the proportion of values in the null-distribution that were equal to, or exceeded, the observed network means.

### Multiple regression and group comparisons

Given that bPLS cannot determine the unique contribution of the variables of interest, we used multiple regression to test the partial association of age and D1DR availability on PLS-derived brain scores. Robustness analyses were carried out to control for auxiliary effects of sex, education, and frame-wise displacement and are reported in full in the supplementary material (S.Table 1). Similarly, multiple regression analyses were used to test the association between brain scores and working memory across the entire sample, controlling for individual differences in age, sex, and D1DR availability. Additional analyses were performed to test for potential differences in association between younger and older individuals, as we have recently demonstrated a shift in the pattern of age-related D1DR differences around 40-years of age[36]. In particular, our prior analysis suggests that age-related D1DR differences in early adulthood (age < 40) may be of distinctive developmental origin, which should not be conflated with D1DR reductions due to aging[36]. We therefore limited our assessment of maintenance to individuals aged 40 and above for group comparisons between high and low levels of D1DR availability as described in the main text, using ANCOVA to test for group differences while controlling for age and sex as covariates of no interest.

## Results

### Spatial patterns of RSFC, age, and caudate D1DR associations

**Fig. 2:**
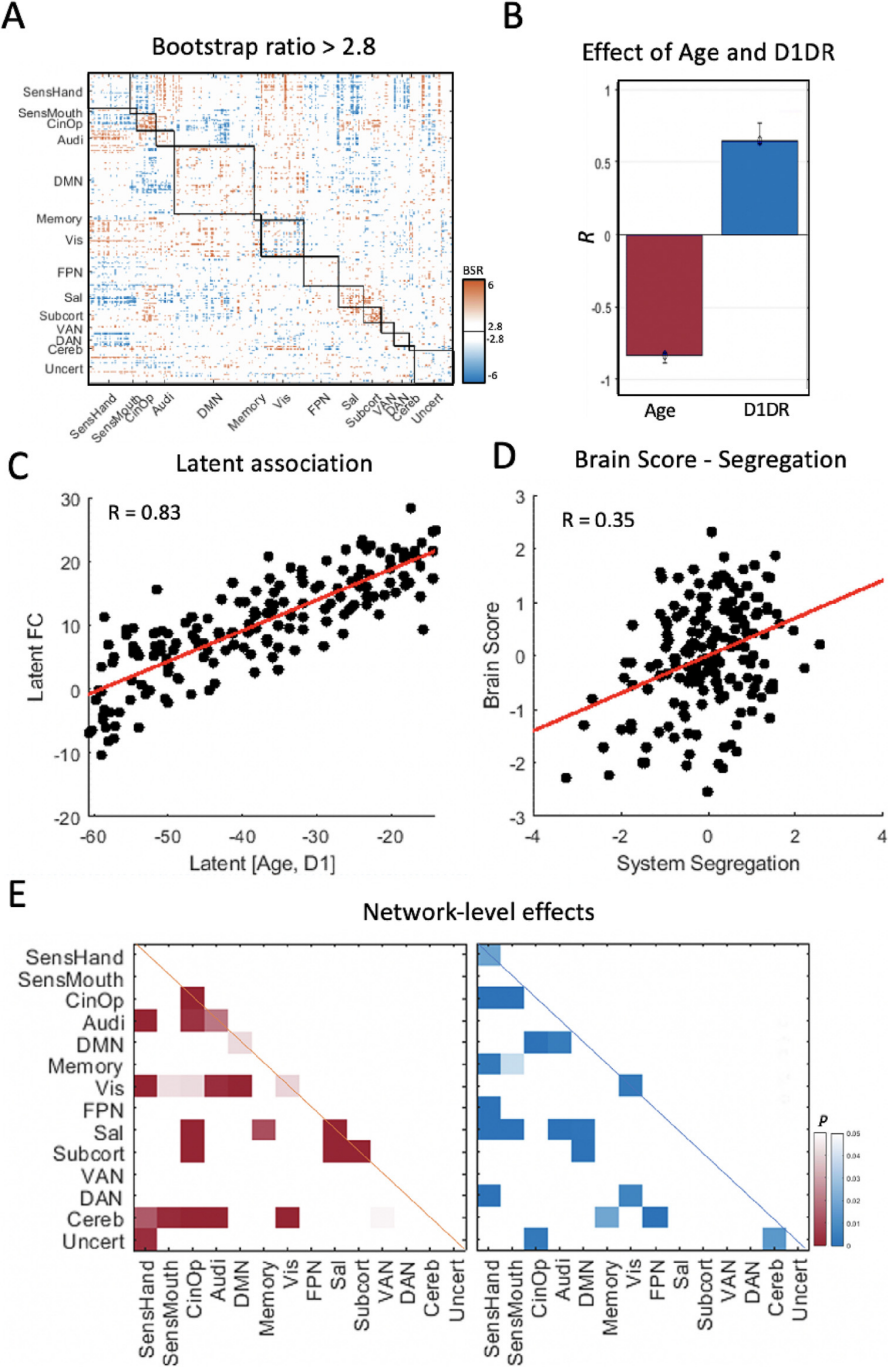
(A) Brain salience matrix depicting significant brain saliences (|BSR|>2.8, *p*≈0.005), representing the spatial pattern of FC edges that are significantly associated with age and caudate D1DR. Warm colors indicate a positive association with the LV and cooler colors indicate a negative association with the LV. (B) Design LV, reflecting the association of age and D1DR in relation to the spatial pattern depicted in panel A. (C) Scatter plot of the latent association between age and D1DR in relation to FC, reflecting the subject-wise partial least squares association between the variables of interest and FC. (D) Scatter plot of the association between system segregation and brain score, indicating a subject-wise correspondence between the spatial pattern depicted in panel A and the degree of segregation between functional networks. (E) Network-level effects of the spatial pattern depicted in panel A, indicating differences in positive (warm colors) and negative (cool colors) associations within and between networks. Color intensities reflect estimated p-values of BSR for each network, with greater intensity indicating a greater association between the variables of interest and FC.

Using PLS analysis, we evaluated the spatial pattern and multivariate relationship between chronological age and caudate D1DR availability on the connectome (Fig. 2a). This analysis revealed one significant LV (permuted *p* < 0.0001). The LV accounted for 91.35% of the cross-block variance and was positively associated with D1DR (latent *r* = 0.65; 95% CI: 0.62, 0.68) but negatively associated with age (latent *r* = -0.83; 95% CI: −0.88, −0.81) (Fig. 2b). Because PLS evaluates the association of D1DR and age in the latent space (Fig. 2c), the partial association of individual variables are not determined. To evaluate the partial association of D1DR and age, multiple regression was used to predict brain score, a metric of how well the PLS-derived pattern is represented in individual subjects. We found that both caudate D1DR availability (R^2^_adj_ = 5.5%, *T*(1 7 0) = 3.15, *p* = 0.002) and age (R^2^_adj_ = 49.7%, *T*(1 7 0) = -12.95, *p* < 0.001) expressed significant partial associations with brain scores, robust to auxiliary effects of sex, education and FD (S.Table 1), indicating that the PLS-derived pattern of connectivity significantly covary with both age and D1DR.

The spatial pattern of significant brain saliences (|BSR| > 2.8) (Fig. 2a) reflect edges that optimally expresses the association between RSFC, age, and D1DR. The pattern indicates that older age and lower caudate D1DR availability is associated with weaker connectivity, particularly within large-scale networks (i.e., positive BSRs). This pattern was evident in several associative networks, including the DMN, cingulo-opercular (CON), salience, and subcortical networks (Fig. 1d). In contrast, an inverse association was observed between several networks (negative BSRs), indicating that older age and lower caudate D1DR availability is associated with greater connectivity, e.g., between the DMN, CON, and auditory networks.

Importantly, the PLS model did not consider any *a priori* demarcation of network structure, nevertheless, the pattern of age-related decreases (and positive link to D1DR) and increases (and negative link to D1DR) were more expressed within and between networks, respectively. Together, this pattern suggests that older age, and lower D1DR availability, is associated with functional dedifferentiation of functional networks. To quantify the degree to which this pattern reflects Age- and D1DR-related dedifferentiation of functional networks, we evaluated the association between brain scores and a measure of system segregation, a metric of the ratio of FC within and between networks[16], [67]. We observed a strong association between brain scores and system segregation (*r* = 0.35, *p* < 0.001), largely unrelated to potential confounds of age, D1DR availability, sex, education, and FD (S. Fig. 1).

## Linking maintenance of D1DR to functional architecture and working memory

Past research have provided ample support for the role of dopamine for efficient working memory function[28], [42], [52], [53], [93]. Moreover, it has been suggested that age-related dedifferentiation of functional networks have negative consequences for cognition at older age [55], [61], [67]. We therefore tested whether the degree of dedifferentiation of age and D1DR-sensitive brain regions are related to working memory performance, and whether maintainence of D1DR at older age is associated with more youth-like connectivity and working memory function (c.f.[64]).

To this end, we used multiple regression to predict working memory performance using PLS-derived brain scores as our independent variable, controlling for age and sex. We expected higher brain scores, i.e., reflecting a more differentiated connectivity structure, to be positively related with cognitive performance. Indeed, a significant association between brain score and working memory was observed across entire sample (*β*_std_ = 0.319, *T*(1 6 8) = 2.966, *p* = 0.003). Post hoc analysis revealed that this association was driven by the older group (Older, n = 117, mean Age ± SD = 50.91 ± 11.26, *T*(1 1 2) = 3.53, *p* < 0.001; Younger, n = 55, Age ± SD = 29.13 ± 5.45, *T*(50) = -1.01, *p* > 0.3).

We recently showed that D1DR differences before and after 40 years reflect developmental and age-related alterations, respectively[36]. To test whether maintainance of D1DR availability at older age is concomitant with greater working memory function and more youthful, i.e., differentiated, network architecture, we restricted our analysis to individuals older than 40 years. This subsample (n = 117, aged 40 – 78 yrs) was further divided into two groups using k-means clustering on age-adjusted D1DR to. Two clusters effectively separated high (*n* = 59, mean D1DR BP ± SD = 1.92 ± 0.17; mean Age ± SD = 61.06 ± 10.73) and low (*n* = 58, mean D1DR BP ± SD = 1.57 ± 0.21; mean Age ± SD = 58.72 ± 11.76) D1DR subgroups, significantly different in mean D1DR (*F*(1,113) = 177.51, p < 0.001). Importantly, the high D1DR-group expressed significantly higher brain scores (high D1-grp: mean ± SD = 7.24 ± 6.94; low D1-grp: mean ± SD = 5.58 ± 6.33; *F*(1,113) = 4.12, *p* = 0.044) and working memory performance (high D1-grp: mean ± SD = 47.48 ± 7.99; low D1-grp: mean ± SD = 46.43 ± 7.89; *F*(1,113) = 5.51, *p* = 0.02). These results indicate that older individuals with greater caudate D1DR avilability also exhibit more youth-like functional architecture, as indicative by higher brain scores, in addition to more efficient working memory processing compared to their age-matched counterparts with lower D1DR availability.

## Discussion

We used PLS analysis to investigate the multivariate relationship between chronological age, caudate D1DR availability, and resting-state functional connectivity. We found that increasing age, and lower caudate D1DR availability, was associated with a spatial pattern of weaker connectivity within networks and greater connectivity between networks. This was particularly evident in associative networks, such as the cingulo-opercular, default mode, salience, and subcortical networks, notably without *a priori* assumptions about network structure in the PLS model. These observations are in line with previous accounts of age-related differences in functional dedifferentiation and segregation[27], [55], [61], [67], [85]. Critically, the inclusion of D1DR availability extends prior work on aging, providing evidence of a dopaminergic mechanism for age-related dedifferentiation in connectivity. Specifically, we found that individual differences in D1DR availability uniquely covaried with expression of the PLS-derived pattern, regardless of subjects’ age. These findings indicate a unique effect of striatal D1DR availability for differentiation of functional systems, in addition to a potential age-related mechanism contributing to increased dedifferentiation in aging.

Although the cross-sectional nature of our study prevents inferences about age-related changes, a possible interpretation is that age-related reductions D1DR density contribute to less distinctiveness of neural representations of the functional connectome. Prior work supports the possibility of a dopaminergic contribution to alterations in network specificity. For instance, in a previous study on individual differences in D1DR availability, Rieckmann et al.[71] demonstrated greater D1DR availability to be positively associated with FC within fronto-parietal network regions, but negatively associated with connectivity between fronto-parietal and default mode regions. Similar findings were reported by Roffman et al.[74], showing that greater striatal D1DR availability was related to stronger decoupling between task-positive and task-negative networks when transitioning from rest to task. While our method was not intended to replicate differences between specific networks, our findings support the notion that reduced striatal D1DR density at older age is associated with less efficient decoupling between networks. In other words, individuals with greater caudate D1DR availability expressed stronger connectivity, particularly between functional systems. Although the exact mechanisms behind D1DR-modulated decoupling of task-positive and negative networks is unclear, and it has been suggested that optimal levels of D1DR enhance task-related and inhibit task-unrelated signaling[46].

Previous studies have shown that maintained functional segregation is metabolically demanding[56], and have positive associations with different neurocognitive functions, such as episodic memory[16], executive function[55], and even global cognition[67]. In line with the previous reports, we found a positive association between network segregation (as indicated by brain score) and a composite measure of working memory performed outside the scanner. This finding, together with brain scores being positively and negatively associated with D1DR and age, respectively, suggests that older individuals with less D1DR show less distinctive large-scale networks and in turn less efficient working memory. Additional analyses comparing groups with higher and lower caudate D1DR availability corroborated that maintenance of D1DR is concomitant with youth-like brain architecture (i.e., higher brain score) as well as more efficient working memory processing. Although an exact mechanistic understanding is lacking, it is possible that optimal D1DR levels exert protective effects against multiple (in)dependent neurobiological cascades in aging (e.g., hallmarks of aging), such as oxidative stress-induced inflammatory responses[86], [97], elevated iron content[32], [77], and white matter hyperintensity[36], [40], [69]. As for the latter, greater white matter lesions at older age have been directly linked to weaker within-network and greater between-network connectivity[38]. Future studies will have to attempt disentangle the relationship between these different factors, and how they contribute to neural dedifferentiation and cognitive aging.

## Limitations and considerations

While the current study provides evidence of a link between striatal D1DR availability and age-related dedifferentiation of functional connectivity, there are some inherent limitations to consider. The network parcellation used in the current work was derived from a younger sample[68], which may have implications for the degree of age-related differences in system segregation. However, it is important to note that our initial PLS analysis does not embrace any specific network assignment (but rather subject-specific 264*264 metric as input). The network assignments based on the parcellation by Power et al. [68] was only used for illustration and evaluation of how well the PLS results reflect age-related decreases and increases in reference to a previously defined network structure. As such, we used an established measure of system segregation, previously been described using same parcellation scheme [16], [67].

Due to the cross-sectional nature of this study, there are some outstanding questions left unanswered. For instance, it is not possible to say whether changes in D1DR is related with dedifferentiation of functional networks. In the same vein, we are unable to say whether greater maintenance of D1DR at older age, i.e., less negative slope of change, has protective effect on network differentiation. It is perceivable that striatal D1DR density is causally linked to decoupling brain regions, yet maintenance of receptor density at old age may not sufficiently prevent dedifferentiation of function. We therefore urge future studies to investigate the longitudinal effects age-related D1DR decline on the functional connectome to disentangle such questions.

## Conclusions

Using PLS analyses, we found that both age and differences in caudate D1DR availability is related to a pattern of neural dedifferentiation in the functional connectome during rest. Importantly, in line with the maintenance hypothesis, we found that greater caudate D1DR at older age was associated with less dedifferentiation of the connectome and greater working memory performance compared to age-matched counterparts with less D1DR. These findings support the notion that age-related deterioration of the dopamine system may have negative consequences for maintaining specificity of functional networks and effective working memory function.

## Declaration of Competing Interest

The authors declare that they have no known competing financial interests or personal relationships that could have appeared to influence the work reported in this paper.
